# Experiences of Continuity of Care Among Registered Nurses Caring for Patients With Chronic Obstructive Pulmonary Disease in Primary Care: A Qualitative Study

**DOI:** 10.1111/jan.16936

**Published:** 2025-04-02

**Authors:** Sara Roos, Malin Sjöström, Jörgen Medin, Christina Melin‐Johansson

**Affiliations:** ^1^ Faculty of Health Sciences, Mid Sweden University Östersund Sweden; ^2^ Department of Public Health and Clinical Medicine Umeå University Umeå Sweden

**Keywords:** continuity of care, COPD, experiences, healthcare professionals, interpretive description, nurses, nursing, primary care, qualitative research

## Abstract

**Aim:**

To explore Registered Nurses' experiences of continuity of care for patients with chronic obstructive pulmonary disease in primary care.

**Design:**

An inductive, descriptive qualitative study.

**Methods:**

Data were collected through semi‐structured interviews with 11 purposively sampled Registered Nurses of varying levels of experience from eight regions in Sweden. The audiotaped interviews were conducted over a 5‐month period (December 2023–April 2024), transcribed verbatim and analysed using interpretive description.

**Results:**

Registered Nurses' experiences of continuity of care for patients with chronic obstructive pulmonary disease are described by three themes (seven subthemes): Patient continuity (Building personal relationships: Being accessible and enabling trust and confirmation), Collaborator continuity (Having a colleague to lean on: Colleagues can lean on me: Feeling alone with my expertise) and Continuity with myself (Trusting my own competence: Carrying a burden alone).

**Reporting Method:**

Consolidated Criteria for Reporting Qualitative Research Guidelines.

**Conclusion:**

This study provides an understanding of Registered Nurses' experiences of continuity of care in primary care. The results may help improve future care since nurses play an essential role in the care of chronic obstructive pulmonary disease within primary care.

**Implications for the Profession and Patient Care:**

To enhance continuity of care for patients with chronic obstructive pulmonary disease, the relationship between the nurse and the patient is important, as is collaboration with colleagues. This collaborative approach allows these nurses to maintain continuity with both the patients and themselves, fostering a more stable and effective care environment.

**Impact:**

This study offers valuable insights into the experiences of Registered Nurses in maintaining continuity of care within primary care, particularly for patients with chronic obstructive pulmonary disease. By highlighting the critical role of Registered Nurses in managing these patients, the study underscores the importance of strong nurse–patient relationships and effective collaboration among healthcare professionals.

**Patient or Public Contribution:**

No Patient or Public Contribution.

**Summary**

- What does this paper contribute to the wider global clinical community?
  ○ This study explores registered nurses' experiences of continuity of care with patients in primary care.
  ○ The nurses strove to build personal relationships with the patients as a framework for continuity. They also maintained continuity with other health professionals and themselves.
  ○ To enhance continuity of care, various factors need to be assessed as time, relationships and knowledge. This applies both to the nurse individually and to collaboration with colleagues and within the primary care clinic. With appropriate support from colleagues when needed, the clinic can serve as a valuable resource within the primary care context where nurses maintain continuity of care with patients.

## 1 Introduction

The primary nursing goal for patients with chronic obstructive pulmonary disease (COPD) is to support them in gaining control over their condition by providing knowledge, ensuring safety and empowering them to prevent exacerbations. According to national guidelines in Sweden, patients with COPD require annual follow‐ups in primary care (PC), with more frequent visits for those who experience exacerbations (National Board of Health and Welfare [NBHW] 2020). Continuity of care (COC) aims to provide high‐quality care when needed (Kohnke and Zielinski 2017), which results in higher patient satisfaction in the long term (Griffiths et al. 2021). Haggerty et al. (2003) defined three different types of continuity. *Relational continuity* is an ongoing responsibility that the healthcare professional, for example, a nurse, has towards a patient over time rather than only during specific periods of illness. This differs from *informational continuity*, which relates to information shared between providers and knowledge about the patient's preferences and values. *Management continuity* refers to shared care management protocols and plans, as well as the flexibility to adapt to changes in the patient's needs. Within PC, the most common continuity type is relational continuity, based on an ongoing relationship that provides patients with a sense of predictability and coherence (Haggerty et al. 2003). Patients in PC who do not have an ongoing relationship with a trusted healthcare professional are less likely to seek help given the lack of continuity and may feel that they do not know where to turn (Tarrant et al. 2014).

## 2 Background

The global prevalence of COPD is about 10.3% (Adeloye et al. 2022; Backman et al. 2020; Global Initiative for Chronic Obstructive Lung Disease [GOLD] 2023), although prevalence estimates may differ due to variations in study designs, diagnostic criteria and analytical approaches (Backman et al. 2020; GOLD 2023). COPD is the third leading cause of death in the world (Aranburu‐Imatz et al. 2022).

In Sweden, healthcare is organised into 21 self‐governing regions. Patients with less severe COPD who do not require hospitalisation are treated in PC, and the regions are responsible for most of the care (Janlöv et al. 2023). Most patients with COPD in PC have a mild to moderate disease grade (Sandelowsky et al. 2016). Some regions have COPD clinics that must be led by an educated asthma, allergy and COPD nurse. To qualify as a COPD nurse, registered nurses (RNs) need additional education in asthma, allergy and COPD, corresponding to at least 15 university credits (Stridsman et al. 2022). At the heart of the nursing profession is the act of caring, and nurses' main responsibilities include promoting health, preventing illness, restoring health and alleviating suffering (Arman et al. 2015). This qualification makes RNs more self‐sufficient when developing care for patients with COPD, allowing them to coordinate and follow up independently (NBHW 2020). A review showed that multi‐competent nurses have high levels of effectiveness. They support patients with COPD in all phases of the disease, improving patient and family empowerment, quality of life, emotional state and pulmonary and physical capacity (Aranburu‐Imatz et al. 2022). Patients are known to value knowledge and support from a trusted nurse concerning their health conditions (Whitebird et al. 2024). RNs are dedicated to caring for patients with COPD. However, they often face challenges in their work because other healthcare professionals sometimes lack competence in managing COPD (Wikman et al. 2024). This can lead to challenges in collaborating with other healthcare professionals (Wei et al. 2022).

Research on RNs' experiences of COC is scarce; COC is mainly described from the perspective of general practitioners (GPs) and their patients (Baker et al. 2020; Cohen and Lindman 2024; Griffiths et al. 2021). Only a few studies describe the role of nurses in COC. Therefore, evidence on the impact of RN continuity on patient outcomes, such as emergency visits, is limited (Bahr and Weiss 2019). RNs are described to be promoting continuity of care, but a more detailed role description is needed (Kobleder et al. 2017). While RNs' experience of COC is not well studied, a few studies have addressed it in the context of heart failure (Östman et al. 2021), midwifery (Hainsworth et al. 2021), surgery (Yakusheva et al. 2017) and managing gaps in COC from a nursing perspective (Jones and Johnstone 2019). Moreover, COC has deteriorated regarding nurse visits for patients in PC; that is, the patients do not see the same nurse to the same extent now as they did 15 years ago (Raivio et al. 2019). To the best of our knowledge, no previous study has exclusively focused on COC and COPD nurses' experiences.

## 3 The Study

### 3.1 Aim

This study aimed to explore Registered Nurses' experiences of continuity of care for patients with COPD in primary care.

## 4 Methods

### 4.1 Study Design

This study was designed as an inductive, descriptive qualitative study with interpretive description (ID), as described by Thorne (2016) and Thompson Burdine et al. (2021). ID was chosen for its exploratory nature, which makes it possible to explore nurses' experiences of COC. Semi‐structured interviews with 11 specialised COPD nurses in PC were conducted. The Consolidated Criteria for Reporting Qualitative Research (COREQ) checklist was used for this study (Tong et al. 2007).

### 4.2 Settings, Participants and Procedure

This study was conducted in Sweden in eight different regions, where specialised COPD nurses care for patients with COPD in PC. RNs in both larger cities and smaller communities from the northern to the southern parts of Sweden were interviewed. From here on, COPD nurses will be referred to as nurses throughout.

Purposive sampling was used by the first author (SR) to recruit RNs with varying levels of experience in caring for patients with COPD in PC COPD clinics (Thompson Burdine et al. 2021; Thorne 2016). The inclusion criteria comprised nurses with at least 1 year of nursing experience working at a COPD clinic in PC, with active involvement in patient care and specialisation as a COPD nurse. Emails were sent by the first author to managers of COPD clinics in various parts of Sweden. However, only three responses were received, and subsequently, no COPD nurses invited this way chose to participate. Most COPD nurses for this study were therefore recruited after the completion of a course in asthma, COPD and allergy which the first author (SR) had participated in. Written information regarding the study was sent by email to all nurses who completed the course and their managers. All RNs worked in different cities and were not colleagues of any of the authors. The potential participants from the course numbered 14, and six agreed to participate. Since this number was too low, snowball sampling was used to recruit more participants (Patton 2015). After each interview, the nurses were requested to ask other RNs if they would be interested in participating in the study. Five other RNs agreed to participate after this. In total, 11 RNs participated in the study.

### 4.3 Data Collection

Prior to data collection, the authors of this study designed a semi‐structured interview guide with open‐ended questions to capture the RNs' experiences of COC. A pilot interview was conducted to help develop the interview questions and determine how to ask them and in what order. The pilot interview was included in the study, and the interview questions were not altered. The pilot interview indicated that the aim of the study was achieved and that the questions were easily understood, and no misunderstandings occurred during the interview. Consequently, no changes were made after this. All data were collected through individual semi‐structured interviews between December 2023 and April 2024 by the first author (SR). The main interview questions were as follows: “Can you tell me what continuity means to you in the care of patients with COPD?” and “How do you experience encounters with patients who have COPD?” Follow‐up questions were asked based on the participants' answers and often included, “Can you tell me more or give an example?” The interviews were conducted either digitally via Teams or Zoom (*n* = 8) or face‐to‐face (*n* = 3) based on the interviewee's preference and lasted 26–71 min (md = 37). The interviews were transcribed verbatim by the first author immediately after completion.

### 4.4 Data Analysis

Data were analysed according to the principles described by Thorne (2016) by the first author (SR) directly after the first interview. A field notebook was also used by the first author (SR) during data collection and analysis to record thoughts, questions and ideas throughout the process. The transcribed interviews were read and re‐read by the first author with guidance from and discussions with the last author (CMJ) to find descriptions that enabled new insights about COC. Relevant sections of texts were marked and described using a few words in the margin. After all the interviews were conducted, the other authors got involved in the analysis. All authors were involved in coding the interview data and discussing different codes and findings until an agreement was reached. The findings were also discussed with other experienced researchers at a seminar, which resulted in further clarification of the findings. As suggested by Thorne (2016), to see beyond the codes, stay true to the study's aim and identify relevant patterns in the data, the questions “What is happening here?” and “What common ideas come up in all or most cases?” served as guidelines. The story that emerged was divided into meaningful themes related to the RNs' experiences of COC. To highlight that the themes and subthemes were grounded in data, the results were illustrated with quotes.

### 4.5 Ethical Considerations

The World Medical Association's Declaration of Helsinki (2024) guided this study. The participants' confidentiality was prioritised. The study received ethical approval from the Swedish Ethical Review Authority (Dnr 2023‐05034‐01) to ensure sample recruitment and data collection and handling followed good research practice. All data were handled and assessed according to the General Data Protection Regulation (GDPR) (Regulation, EU 2016). The participants and their managers received oral and written information about the study, as well as the opportunity to withdraw from the study at any time. Written informed consent was obtained from each participant and their manager before the interview, along with their consent for audio recording. The participants decided the time and place for the interview because their integrity and self‐determination were highly valued during the study's implementation. All transcripts were numbered with a code, and any information that could potentially enable identification of the participants or patients was removed. All transcripts were stored on the authors' computers' private catalogues (according to university policies) and secured with personal passwords.

### 4.6 The Researchers' Pre‐Understanding

The first author's (SR) pre‐understanding included experiences from working in a COPD clinic in PC as a COPD nurse. All participants were informed about SR's professional background, and six participants knew SR from the course in asthma, allergy and COPD. Of the co‐authors, CMJ and JM are both RNs, and MS is a GP. All are experienced researchers. Reflecting on the authors' pre‐understandings helped navigate potential preferences and different views more effectively.

### 4.7 Rigour and Reflexivity

Rigour was maintained by using Thornes' (2016) criteria of quality that include the following: (i) *Epistemological integrity*: Co‐authors' guidance and feedback were used in this study; (ii) *Representative credibility*: The sample included nurses from eight regions and with different levels of experience. This can be seen as an important key instead of saturation power; hence, a wider range of participants were included in the study; (iii) *Analytical logic*: An audit trail was used in this study to help with reflections and understanding of the dataset; and (iv) *Interpretive authority*: Transcripts were prepared verbatim by the first author. All authors met regularly to discuss their coding progress and to explore and share ideas. At these meetings, transcripts, notes and coding were shared. According to Thompson Burdine et al. (2021), themes develop during the process of the authors familiarising themselves with the data. The authors discussed the identified themes frequently. Through this ongoing process, the themes in the dataset began to emerge, and the authors then discussed and presented them in the findings below.

## 5 Findings

### 5.1 Characteristics of the Study Participants

The participants included 10 females and 1 male. All had over 10 years of experience as RNs, with 6 of them having over 20 years of nursing experience. Their tenure at the current COPD clinic ranged from 1 year to over 10 years. All nurses were over 35 years old, and 3 were 55 years or older.

### 5.2 RNs' Experience of COC for Patients With COPD

**FIGURE 1:**
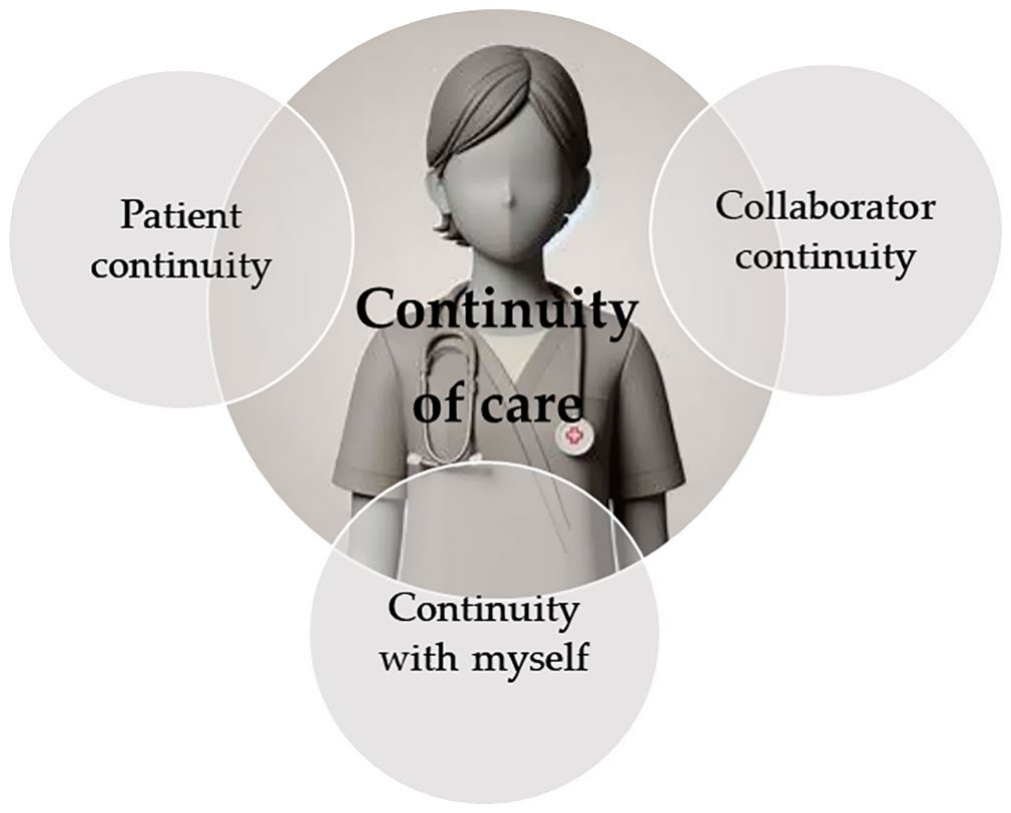
The three themes developed from the analysis.

The analysis of interviews led to the development of three themes: *Patient continuity, Collaborator continuity* and *Continuity with myself* (Figure 1) and seven subthemes (Table 1).

**TABLE 1:** Themes and subthemes.

Patient continuity	Collaborator continuity	Continuity with myself
Building personal relationships	Having a collegue to lean on	Trusting my own competence
Being accessible and enabling trust and confirmation	Collegues can lean on me	Carrying a burden alone
	Feeling alone with my expertise	

These three themes and seven subthemes describe the RNs' experiences of COC when caring for patients with COPD.

The themes need to be understood in the context of PC in Sweden. From here on, RNs are referred to as nurses.

### 5.3 Patient Continuity

The theme is described with two subthemes: *building personal relationships* and *being accessible and enabling trust and confirmation*. The theme refers to the RNs' desires to create a long‐lasting relationship with the patients and their next‐of‐kin by acknowledging the person behind the disease and striving to fulfil their aims for treatment and nursing care, letting the patient set the tone.

#### 5.3.1 Building Personal Relationships

The nurses reported that while a relationship can be initiated at the first meeting, ongoing interactions are essential to build a strong personal connection over time. Despite challenges, such as patients feeling unwell or being unwilling to make lifestyle changes, the nurses experienced the meetings as often being successful. The patients also became more motivated to turn to the nurse for help as the relationship matured and became personal.

Such a relationship is difficult to build without continuity because it is as if over time, you gather knowledge about the patient, both how they are and how they are purely symptomatic, what symptoms they have and what type of exercise they do and how they live. This is something we can use for the next meeting, something to talk about, so this is good. (P1)

When the nurses dedicated time to patients, they focused on a person‐centred approach, encouraging the patients' narrations to lead the conversation. This helped the nurses to understand the patients' perspectives, such as reasons for non‐adherence to prescribed treatments, and to work collaboratively to find acceptable solutions. Asking simple questions like “How are you?” or “How do you feel?” fostered a caring relationship where the patients felt listened to. The nurses adapted their communication to each patient, even using dialects when appropriate. Taking time to engage on a deeper level with patients enabled the nurses to better understand their needs, which made the work more rewarding. The nurses stated that trust‐building was essential, particularly as the patients may feel ashamed of their disease. This required attentive and supportive nurses to address the patients' emotional and medical needs.

Most often, a large part of the meetings can be spent talking about a dead spouse or other things, you notice that there are many other needs. So hard to catch up with what you must do, I think, sometimes, according to the guidelines, and so you must be a little pragmatic and see what you have in front of you. Then you can book a follow‐up visit instead. (P9)

Additionally, some patients required more frequent visits with the nurse than others. Without the ability to follow up on these visits, there would be no continuity or relationship with the patient. Next‐of‐kin also appreciated the continuous contact patients had with the nurse, such as when family members were familiar with the nurse and felt confident that the patient was well cared for. This was especially reassuring in cases where the patient was unable to talk with the nurse independently due to hearing difficulties or loss of other physical functions.

#### 5.3.2 Being Accessible and Enabling Trust and Confirmation

Being accessible and ensuring that the patients can reach the nurse at any time is important for COC. The patients could call the nurses at a specific phone number for 1 h every day. This made the patients feel safe, knowing that they could reach the nurse if they felt unwell. The nurses could also receive messages from their colleagues if patients called the main number of the PC and asked to speak with a certain nurse. The nurses also often left the door to their office open so that patients could get in touch with them in person when needed.

My patients can drop by and mention that there's a special offer on a pillow at a department store, or they might want to check their oxygen saturation level. They want to check in and get some reassurance that I still think they seem to be doing quite well, and that's the advantage of me being quite accessible. They can come by, so they don't need to be acutely ill to seek me out. (P2)

Conversely, patients who were not accustomed to receiving regular care from a nurse rarely sought care, thus tending to delay seeking help until the last minute. Being accessible made patients aware that a COPD nurse works at the clinic and helped them seek care earlier and more appropriately.

Identifying me as a COPD nurse who is always available created trust. (P11)

Trust was created when patients could reach the nurse in serious situations, for example, when the patient was anxious, which might lead to shortness of breath. A nurse who listened and assisted with resistive breathing could prevent the need for an ambulance and an emergency visit. To achieve this, the nurse must instil a sense of safety and trust in the patient.

Continuity is the most important thing to gain trust between the patient and the nurse, that they know that I am here if they need me. If you show genuine interest and not only doing your job, but you also show engagement, then the treatment goes so much better for the patient. (P9)

The nurses supported the patients' need to feel safe during an exacerbation. To make the patients secure in themselves and feel able to cope with situations, the nurses needed to be outspoken and repeat information at every appointment. To make the best of the situation, the nurses needed to customise the information to each patient.

If I can help one patient gain knowledge about their condition, understand what to do in all situations, and feel secure with us, knowing they can reach out whenever needed, then I have succeeded. (P5)

### 5.4 Collaborator Continuity

The second theme, Collaborator continuity, is described with three subthemes: *having a colleague to lean on, colleagues can lean on me* and *feeling alone with my expertise*. The theme refers to the RNs' experiences of collaboration or lack thereof within PC, that is, with the GPs and other healthcare professionals.

#### 5.4.1 Having a Colleague to Lean on

Nurses who worked with another COPD nurse at the same clinic aimed to meet the same patients to ensure continuity. This approach allowed them to build relationships with the patients and discuss previous interventions or progress. They divided the responsibility for patients between them to effectively manage patients' care needs. This collaboration fostered a supportive environment where the nurses could share knowledge and address specific challenges related to the patients' needs with each other. Here, continuity served as a safety net, ensuring that the nurses did not feel isolated in their profession regarding both their patients and their colleagues.

We talk every day, above all about the spirometry, which is difficult to interpret and so on, and then a lot about counselling support, for example, do you think I thought correctly here? (P8)

Nonetheless, COC does not only include a single nurse but is more of a consensus with colleagues regarding patient care. Thus, it was not only the nurse's responsibility to create continuity with the patients, but also the duty of the whole team of GPs, COPD nurses, physiotherapists, dietitians and the manager. Care was more flexible when GPs were interested in the care of patients with COPD, but this varied. Good cooperation and continuity with doctors were beneficial for the nurses' management of patient care. However, this was much harder to achieve if doctors only worked temporarily at the clinic. Although GPs rarely met patients with COPD, the collaboration between nurses and GPs was successful, and everyone knew their role. The nurses perceived that the clinic bridged the gap between patients and other professionals.

These COPD patients rarely seek the doctors since it is us—the nurses—that see them, and we let the doctors know when there is something special, they need to do. We run the COPD clinic with support when needed from the doctors. (P7)

#### 5.4.2 Colleagues Can Lean on Me

The nurses' competence regarding COPD was a resource in PC, and colleagues could consult them about inhalation techniques or discuss specific patients. The GPs sometimes asked the nurse to meet with the patient before prescribing medication. The nurses also met patients newly diagnosed with COPD and were trusted by GPs to explain the stage of the illness and recommended medications.

I gain a lot of confidence from the doctors that I have a significant influence on how we proceed. I can also recommend performing X‐rays, treatments, and so on. There, I receive a great response. (P11)

COC also included the medical treatment the patient received. It was easier for the nurses to understand why a patient did not take the prescribed medication when they knew how their patients thought or considered that the inhaler might be too difficult for the patients to use. Providing correct information and knowledge about the patients' medical treatment was important to preserve continuity. The nurses expressed that GPs sometimes had trouble knowing what treatment and inhaler would be most suitable for the patients. Here, the nurses' knowledge was valuable, and they could follow up on the prescribed medication and ensure COC. The nurses would call the patients after a few months to ensure that the prescribed medication was working for them, and sometimes, they would ask the patients to return for another round of spirometry to evaluate the medication.

Ensuring that they take their medications, monitoring so that they don't…I mean, preventing various deteriorations of any kind. It's so important to have continuity. (P4)

#### 5.4.3 Feeling Alone With My Expertise

The nurses often felt alone with their expertise and that they did not have anyone to share their thoughts and worries with, neither with another COPD nurse nor with a GP. The nurses also expressed a substantial sense of responsibility towards the patients. They often went beyond their official duties and available resources to ensure comprehensive care, arguing that it is better for patients to be cared for by someone who knows them. Providing COC alone made the nurses vulnerable and often responsible for all actions, from providing information to making difficult medical decisions.

It may be the case that we inhale them to absurdity sometimes. I feel that we make them inhale before lunch, and then we administer another round to see what happens. After a few hours, they come back, and then we make them inhale again instead of sending them to the hospital and getting them admitted. (P2)

The nurses considered it valuable to discuss medical issues with a GP who knows the patient. However, sometimes, only relay GPs were available, or the doctors were not engaged with the patients' problems. Under these circumstances, the nurses felt abandoned, and the lack of support made the situation difficult to handle.

One thing I miss is that there is a lack of continuity with the doctors when it comes to care for patients with COPD. Because then it's me who is the continuity, so to speak, and maybe that's a good thing. I think the patients see a lot of different GPs, but some doctors also just let the COPD care go and think this is a matter for me as the COPD nurse. (P6)

### 5.5 Continuity With Myself

The third theme, Continuity with myself, is described with two subthemes: *trusting my own expertise* and *carrying a burden alone*. The third and last theme describes the RNs' appreciation for continuity with themselves as a nurse, for example, the importance of reading the notes in the patient's records and its value in helping them to maintain continuity in care. However, COC with some patients can be a burden, and RNs may feel alone.

#### 5.5.1 Trusting My Own Competence

The nurses trusted their own competence and adapted the follow‐up accordingly. They used structured treatment plans as a guide in planning patient care, with the patients' needs and concerns forming the core. For example, the nurses assessed if their patients needed appointments more frequently than once a year based on each patient's medical history with exacerbations or other concerns that might need more attention. One nurse said that she observed a patient on the street who had trouble breathing at the time, and she could arrange for a prompt follow‐up and help the patient before the situation became critical.

I see them out in the community panting like a moose, so I can call them for a follow‐up that much faster because I know who it is. (P2)

Establishing the COPD clinic was highly rewarding for the nurses and enhanced their confidence through familiarity with their patients. They emphasised that COC involves the ability to follow up with their patients, reviewing previous decisions and actions. By examining their notes and medical records prior to appointments, the nurses stayed informed about past discussions and planned follow‐ups accordingly. Continuity was described as crucial for detecting changes between appointments, ensuring that patients did not need to repeat the same information constantly.

I can recall that the last time you were here, you weren't as short of breath when you left the waiting room. What has happened since then? (P1)

#### 5.5.2 Carrying a Burden Alone

Not being able to build a trusting relationship with a patient was described by the nurses as carrying a burden alone; in such cases, continuity was a barrier rather than an asset. If an appointment did not end well, it could lead to the patient not returning to the clinic because their trust in the nurse was lost, or they felt that they did not work well together. Such occurrences make patients vulnerable and alone, without anyone to whom they can turn. Here, continuity is not an advantage but a burden, both for the patient and the nurse, and the nurse might need to take a step back and suggest that they start all over again.

Sometimes it just doesn't get right. Then, continuity can be a burden when you can't leave this to a colleague. But this rarely happens since I usually adapt the meetings to who I have in front of me. (P11)

The nurses sometimes felt like they were carrying a burden alone and that they lacked support from their colleagues. For example, medical issues other than COPD might cause problems that are challenging to handle. The nurses also described that some patients who continued smoking and did not want to quit demanded additional care and support, which could be difficult to provide. It was discouraging to be unable to do more for these patients, especially when they lacked insight into their illness.

They continue to smoke, and the lung clinic does not accept them because they do understand that they need to quit smoking, but they are unable to do so. (P11)

The nurses acknowledged that even though there was not much they could do for such patients, the patients kept coming to see them since there was an established continuity between the nurse and the patient, and the patients felt some trust.

## 6 Discussion

This study aimed to explore Registered Nurses' experiences of continuity of care for patients with COPD in primary care. The most important finding was that continuity is valued by nurses in the context of building personal relationships with patients and determining their needs. The nurses also described continuity with colleagues and with themselves in their professional roles as important. The themes and subthemes that emerged highlight how complex and challenging it can be to maintain continuity when caring for patients with COPD and how it may affect the nurses. The three themes and their subthemes are discussed below.

Regarding the theme Patient continuity, we found that *building a personal relationship* with the patient included gathering knowledge over time to establish a relationship, such that the nurses get to know their patients as individuals with specific problems. This aligns with Haggerty et al.'s (2003) description of relational continuity. On the contrary, Östman et al. (2021) stated that nurses who cared for patients with heart failure did not perceive a caring relationship as needing to be personal. In their study, the nurses considered having someone to turn to the most important factor, not a specific nurse. In the present study, the nurses highly valued personal relationships and found them key in building trust between the patient and the nurse. This may be related to the role of the COPD nurse since most of the time, there are one or two nurses at every COPD clinic, increasing the importance of supporting the relationship. In this study, the nurses also stated that time was essential to building a good personal relationship. Having enough time enabled patients to talk about private matters, such as the death of a spouse. Such meetings strengthened the relationship between the patient and the nurse and forged bonds. Ljungholm et al. (2022) also stated that time is essential, and if patients are given the space to talk freely, mutual goals are easier to achieve.

In the present study, nurses considered it important to let the patients set the tone of the encounters and outline the consultation accordingly. The nurses worked based on a person‐centred care approach to *building personal relationships*. This helped the nurses to understand how their patients thought when they were not compliant with medical ordination or did not use their inhalers. Engaged relationships and a person‐centred care approach are key attributes of relational continuity (Bahr and Weiss 2019; Haggerty et al. 2003). According to Bahr and Weiss (2019), nurses identified themselves as knowing the patient both personally and clinically and considered this essential to their ability to provide the correct care for each patient while meeting their personal needs. This sentiment was echoed by all COPD nurses in the present study. According to Arman et al. (2015), care should be understood from the perspectives of patients and their next‐of‐kin, and our study shows that COPD nurses strive to fulfil this requirement. The theory of person‐centred care provides practical and ethical guidance to build relationships between healthcare professionals, patients and their next‐of‐kin. The foundation of the theory values respect for the person, the person's right to self‐determination and understanding. To achieve this, a care culture that strengthens the staff and creates a prerequisite for continuous development in the work field is needed (McCormack et al. 2021; McCormack and McCance 2006).

In the theme Patient continuity, the subtheme *being accessible* as a nurse highlighted the significance of providing care to patients, especially those who had not been properly followed up or monitored previously in PC. Patients had “fallen through the cracks”, as described by Tarrant et al. (2014), or had not been taken seriously even though they had a chronic disease (Gjengedal et al. 2019), until the introduction of the COPD nurse, who now maintains COC for such patients.

Regarding the theme Collaborator continuity, we found the subtheme *having a colleague to lean on*, which included having a supportive manager or colleagues in the COPD clinics. When they had someone to rely on and trust, nurses' feelings of support increased, and they felt that they did a good qualitative job, which is the core in caring for patients with COPD. On the other hand, nurses who lacked this kind of support often felt that their responsibilities towards the patients could become overwhelming. Such nurses might *feel alone with their expertise*, not having anyone to turn to when needed. This was also found by Haggerty et al. (2003), who described informational continuity as a continuous sense of responsibility. According to Östman et al. (2021), nurses often take on the main responsibility for patient care, but they need to network with other professionals to provide the best possible care. Management continuity refers to the whole care team, wherein the nurse is part of the coordinated care, but the whole team, including GPs, takes responsibility (Bahr and Weiss 2019). Moreover, good management continuity is particularly important for chronic diseases such as COPD, where several providers might be involved (Haggerty et al. 2003). However, Wikman et al. (2024) identified limited competence as a barrier to collaboration. The reduced knowledge about COPD among other professions may hinder interprofessional collaboration in managing COPD care. Lack of routines and structure, along with unclear distribution of responsibilities, was also identified as barriers to collaboration (Wikman et al. 2024). Nonetheless, when interprofessional collaboration is effective, it has positive effects on healthcare professionals, as noted by Wei et al. (2022). This aligns with the results of this study.

In the theme Continuity with myself, the COPD nurses explained that they reviewed the patient's medical records before the encounters to stay updated and informed on previous discussions, and this step was even more valuable when they read their own notes. Ljungholm et al. (2022) showed that together with the ability to listen and build trust, the possibility to read patients' medical records in advance is a huge advantage in achieving continuity. Bahr and Weiss (2019) also found that good informational continuity allows nurses to make care decisions based on an accurate picture and timeline of past events. Another way of maintaining continuity in our study was found in the subtheme *trusting my own expertise*, where the nurses used structured treatment plans in their efforts towards continuity. According to Haggerty et al. (2003), treatment plans are valuable for maintaining management continuity, but documented information is also seen in informational continuity, where knowledge about the patient's preferences and values answers to the patient's needs. The nurses in the present study labelled treatment plans as a valuable tool that helped them to follow up on the patients' goals and agendas and were important when evaluating events since the last encounter. These structured treatment plans can be seen as a mutually agreed‐upon health plan, which helps the nurse to adapt the care in dialogue with each patient (Ekman et al. 2021). The importance of treatment plans has also been emphasised in studies by Jones and Johnstone (2019) and Östman et al. (2021); however, they are sometimes difficult to complete for all levels of care (Östman et al. 2021).

### 6.1 Strengths and Limitations of the Work

Although COC is an international phenomenon, this study was limited to a Swedish care context, which may affect the transferability of its findings to other clinical contexts. The readers need to consider this and evaluate if the results are applicable to other contexts. Another limitation is that the COPD nurses who agreed to participate in this study were all very engaged in their work and wanted to do a good job. Thus, they might demonstrate more commitment to their work than others, and a more positive vision might be the result. Since the first author (SR) was slightly acquainted with six of the interviewees, this might influence the findings. The respondents might have wanted to present their experiences in a more positive light, given that they and the first author had participated in the same course. This was also discussed in the research group, but nothing arose during the process that required further clarification. The interviewees spoke freely, and there were no differences in depth and information between those who knew SR and those who did not. None of the authors were coworkers with the interviewees.

To minimise the influence of the interviewer's pre‐understanding on the interviews, a semi‐structured interview guide with pre‐arranged questions was used, and a pilot interview was conducted. The first author (SR) is a PhD student with little prior training, but the co‐authors are experienced researchers, and all interviews were discussed after data collection to confirm that the interview questions allowed the participants to talk freely about their experiences. This included determining if something was missing or needed to be followed up on during the interview. Ongoing reflection during the process included the researchers' understanding as themes emerged. The field notebook was also a helpful tool for the first author (SR) during interviews and data collection for reflecting on thoughts, questions and ideas.

Distance and costs may justify the use of digital interviews, as the breadth and quality of data are comparable between face‐to‐face and digital interviews (Krouwel et al. 2019). In this study, both face‐to‐face and digital interviews produced the same amount of data. There were no differences in the duration of the interviews, with the longest interview (71 min) being a digital one. Most of the participants work in other cities, sometimes as far as 800 km or more from the research group. Consequently, digital interviews were the only feasible method to conduct the interviews in most of the cases in this study.

One strength of this study is that COPD nurses from different clinics and regions in Sweden were included, which makes the results transferable to other nurse specialist clinics within the country. Moreover, both small and large COPD clinics were included. Additionally, all the nurses had completed a university course in asthma, COPD and allergy and were experienced nurses, having cared for many patients with COPD over various periods. Among the COPD nurses in this study, 10/11 were female (91%). According to the NBHW (2024), in 2023, 87% of nurses in Sweden were female, so this distribution aligns with national data.

### 6.2 Recommendations for Further Research

A deeper understanding of patients' experiences of COC is recommended. Is there a consensus between the nurses' and patients' experiences and understanding of the value of nurse‐led COPD clinics? Continuity may be valued differently by various patient groups, but it has not been considered fully in this context according to patient satisfaction and overall care quality. Another topic of interest is exploring GPs' perspectives on COC for patients with COPD.

### 6.3 Implications for Policy and Practice

Three different aspects of COC need to be considered: COC with the patient, COC with colleagues and COC with the nurses themselves. According to our results, the best care for COPD patients was provided when nurses worked independently with good support from colleagues and GPs when needed. Having a colleague to lean on made the nurses feel more secure and meant that they, in turn, could support their colleagues while adapting care for each patient. To further enhance patient care, it is essential to clarify the routines for when other healthcare professionals, such as GPs, need to take on more responsibility. These challenges may stem from a lack of organisational support. To address this, organisations need to expand interprofessional collaboration and allow staff the time to develop care.

## 7 Conclusion

This study indicates that COC for patients with COPD is highly valued among nurses in PC. Building personal relationships is the key to learning patients' needs and helping them effectively. If time and resources are available to build COC, high‐quality care is provided. Nurses who can manage their practice with support from managers and colleagues if needed provide a competent and important job for everyone involved. Such nurses thrive, develop an independent practice and form the core of COPD care in PC. To achieve this, nurses need to be determined and appreciate their responsibility since they can sometimes feel lonely in their position. This study adds to the evidence that nurse‐led COPD clinics are meaningful and valuable models for nurses to follow since, in this context, they believe that their work is both important and fulfilling.

## Ethics Statement

The study received its ethical approval from the Swedish Ethical Review Authority (Dnr 2023‐05034‐01) to ensure that sample recruitment, handling of data, and data collection ensure good research practice.

## Conflicts of Interest

The authors declare no conflicts of interest.

## Data Availability

Data is available on request due to privacy/ethical restrictions.
